# Fibrinogen as a Key Binding Partner in Human Plasma for Lecanemab Biosimilar IgG

**DOI:** 10.1002/alz.095317

**Published:** 2025-01-09

**Authors:** Jean‐Pierre Bellier, Andrea M. Román Viera, Caitlyn Christiano, Juliana A. U. Anzai, Stephanie Moreno, Emily C. Campbell, Lucas Godwin, Amy Li, Alan Y. Chen, Sarah Alam, Adrianna Saba, Han bin Yoo, Hyun‐Sik Yang, Jasmeer P. Chhatwal, Dennis J. Selkoe, Lei Liu

**Affiliations:** ^1^ Brigham and Women’s Hospital, Boston, MA USA; ^2^ Massachusetts General Hospital, Boston, MA USA

## Abstract

**Background:**

Therapeutic monoclonal antibodies targeting amyloid beta‐protein (Ab), such as lecanemab, represent a promising approach for disease‐modification in Alzheimer’s disease (AD). Due to its relatively short half‐life, lecanemab is given as a bi‐monthly infusion (typically 10mg/kg). Binding to high abundance plasma proteins (PPB) can influence the pharmacokinetics of drugs in the blood, including their half‐life.

**Method:**

In this study, we investigated the potential of PPB for lecanemab using a biosimilar synthetized from publicly available lecanemab sequence (Lec‐bs). Lec‐bs biosimilar immunoreactivity was assessed in human plasma fractions obtained by size exclusion chromatography using both ELISA and Western blotting. The binding of lec‐bs to candidate PPB was validated through Western blotting, ELISA, and surface plasmon resonance analysis.

**Result:**

Using a combination of equilibrium dialysis, ELISA, and Western blotting of human plasma, we first describe the presence of likely PPB for Lec‐bs, and then identify fibrinogen as a probable major plasma protein binding partner for Lec‐bs. Utilizing surface plasmon resonance, we confirmed that Lec‐bs binds to fibrinogen, albeit with lower affinity (318 µM) than to monomeric Ab (324 nM).

**Conclusion:**

In the context of lecanemab therapy, these results suggest that fibrinogen levels may influence free antibody levels in blood and that fibrinogen may act as a reservoir for lecanemab. More broadly, these results indicate that PPB may be an important consideration for the clinical use of therapeutic antibodies in neurodegenerative disease.

